# Unilateral Focused Ultrasound-Induced Blood-Brain Barrier Opening Reduces Phosphorylated Tau from The rTg4510 Mouse Model

**DOI:** 10.7150/thno.28717

**Published:** 2019-07-13

**Authors:** Maria Eleni Karakatsani, Tara Kugelman, Robin Ji, Maria Murillo, Shutao Wang, Yusuke Niimi, Scott A. Small, Karen E. Duff, Elisa E. Konofagou

**Affiliations:** 1 Department of Biomedical Engineering, Columbia University, New York, NY 10032, USA; 2 Taub Institute for Research on Alzheimer's Disease and the Aging Brain, Columbia University, New York, NY 10032, USA; 3 Department of Neurology and Pathology, Columbia University, New York, NY 10032, USA; 4 Department of Pathology & Cell Biology, Columbia University, New York, NY 10032, USA; 5 Department of Integrative Neuroscience, Columbia University, New York, NY 10032, USA; 6 Department of Radiology, Columbia University, New York, NY 10032, USA

**Keywords:** focused ultrasound, blood-brain barrier opening, tau pathology, Alzheimer's disease

## Abstract

The neuropathological hallmarks of Alzheimer's disease include amyloid plaques and neurofibrillary tangles. Tau pathology correlates well with impaired neuronal activity and dementia. Focused ultrasound coupled with systemic administration of microbubbles has previously been shown to open the blood-brain barrier and induce an immune response, which, in an amyloid AD mouse model, resulted in the reduction of the amyloid brain load.

**Methods**: In this study, we investigated the effect of focused ultrasound at the early stages of tau pathology (pre-tangle) in the rTg4510 mouse model.

**Results**: Reduction of phosphorylated tau from the hippocampal formation processes, and particularly the pyramidal CA1 neurons, was noted in the ultrasound-treated brains without an associated increase in the phosphorylated tau-affected cell somas, typically associated with disease progression. Attenuation of the pathology was found to correlate well with the ultrasound-initiated immune response without compromising neuronal integrity. Unilateral ultrasound application resulted in a bilateral effect indicating a broader reduction of the phosphorylated tau.

**Conclusion**: Findings presented herein reinforce the premise of ultrasound in reducing tau pathology and thus curbing the progression of Alzheimer's disease.

## Introduction

Alzheimer's disease is a chronic neurodegenerative disorder characterized histopathologically by the accumulation of insoluble forms of amyloid-β (Aβ) in plaques and the aggregation of hyperphosphorylated forms of the microtubule-associated protein-tau (p-tau) into neurofibrillary tangles in neurons 1,2. The existing literature indicates that these two hallmark lesions, and the accumulation of toxic intermediates from which they are formed, are implicated in the initiation and progression of dementia. The amyloid cascade hypothesis 3 suggests Aβ promotes tauopathy which has been demonstrated in numerous experiments. The pathological cascade of events that leads to tauopathy and neurodegeneration includes tau hyperphosphorylation, misfolding and aggregation, destabilization of the microtubule network and the cytoskeleton, deficits in axonal transport, synaptic loss, neurodegeneration and cell death 4,5. Tauopathy has been shown to propagate between brain regions, and it can undergo trans-cellular transfer either from the somatodendritic compartment or at the axon terminals across the synapses 6. The mechanism of transfer is not known but it is likely vesicle mediated or via the release and uptake of conformers that act as prion-like seeds 6,7. Biomarker association with disease progression places tau accumulation at the earliest stages of dementia, while the density and spatial distribution of hyperphosphorylated tau have been linked to cognitive decline 4-6,8, thus making tau an attractive therapeutic target 9.

From clinical standpoint, the cornerstone in prevention and management of Alzheimer's disease is the deceleration of the progression through non-pharmacological approaches -behavioral techniques and adjusted nutrition- and pharmacological agents targeting cognition enhancement and systemic complications management 1. Clinically, several phase III trials have been performed in patients with mild-to-moderate Alzheimer's disease but failed to show significant improvement in their primary outcomes 10,11. It was only until four years ago that the phase 1b clinical trial administering aducanumab in Aβ PET-positive patients (prodromal or mild AD) reported on reduced levels of Aβ in a dose- and time-depended regime, yet the trial was not valued for the clinical endpoints given the observational nature of the data and the missing corrections in the statistics 12. Finally, the emerging technology of focused ultrasound (FUS) was recently evaluated in terms of its clinical safety and feasibility in five early-to-moderate Alzheimer's patients, succeeding in inducing a transient blood-brain barrier (BBB) 13 opening without adverse effects 14.

The smooth transition of this technology to clinical applications could be attributed to the extensive preclinical validation of FUS in experiments involving the safe, localized and reversible opening of the BBB 15-18. The mechanism of BBB disruption with FUS is not entirely clear. It has, however, been proven, that the interaction of systemically-injected microbubbles with the microvascular walls is the main driving mechanism of the technique 19,20. The BBB can be mechanically disrupted by cavitational activity undergone by the oscillating microbubbles that enter the focal region of the ultrasound beam during circulation 21, thereby engaging the surrounding capillary and arteriole walls and thus loosening the tight junctions 19-22.

FUS has been evaluated in experiments involving the safe BBB disruption of various animal species including rodents, lagomorphs and primates 15,23-25 and in different disease-mimicking models 26-29. The integrity of the BBB is restored within hours and remains intact 17 depending on the ultrasound parameters 25. FUS-induced BBB opening was initially introduced as a surrogate to drug delivery techniques that fell short in efficaciously surpassing the hindrances posed by the BBB hence their invasive and/or non-targeted nature 30. Over the recent years, compounds of varying molecular weights have been successfully delivered to the brain parenchyma, including contrast agents 17, antibodies 31, chemotherapeutics 32 and neurotrophic factors 33,34.

Specifically in Alzheimer's disease, antibodies against Αβ 31,35 have been mediated into the brain parenchyma by FUS, which resulted in the amyloid plaque load reduction. More importantly, these studies showed that amyloid could be cleared even in the absence of the antibody indicating the immense potential of FUS-facilitated BBB opening as an immunomodulatory stimulant. Transcriptomic analysis of the FUS-induced bioeffects outlined the profile of the triggered acute inflammation. Aside from forming an environment that could favor neuro- and angiogenesis, FUS-induced BBB opening was proven to contribute to the clearance of pathological proteins 36 accounting microglia activation as part of the response 26,31.

The role of microglia in neurodegenerative diseases remains a challenging topic. Although microglia respond immediately to acute injury, morphologically activated microglia for an extended period of time fail to synthesize inflammatory mediators that could negatively impact neuronal integrity 37. Prolonged activation of microglia is present in chronic neurodegenerative disorders, yet efforts to inhibit inflammatory mediators secreted by microglia failed to ameliorate the degeneration 37. In Alzheimer's disease, microglia acquire an “activated” phenotype yet fail to reduce amyloidosis 38, while the link between microgliosis and tau pathology remains to be established 39,40. Stimulus-dependent conformational changes and dystrophic microglia have been associated with tau pathology, while re-programming of the microglia to healthier phenotypes has recently emerged as a therapeutic strategy 41,42.

Whether FUS-induced BBB opening using a safely tolerated paradigm 26,31 could drive an immune response characterized by the activation of microglia 43, and/or infiltration of peripheral immune cells 25,43,44 such as macrophages and monocytes to help ameliorate such pathologies, remains unclear but these mechanisms could explain the positive effects observed. Recently, a decrease in phosphorylated tau from the entorhinal cortex in a human-tau mutant transgenic mouse strain (line pR5) has been reported following antibody delivery by repeated FUS-mediated BBB opening over the entire brain 28. However, the interaction of FUS with tau pathology has not been studied from the immunotherapeutic angle.

We have now examined whether early-stage tau pathology can be attenuated by ultrasound to (i) investigate the efficacy of unilateral FUS, as an adjuvant treatment, for the reduction of phosphorylated human tau in the hippocampus of the rTg4510 mouse model of AD and (ii) identify the response of the FUS-initiated immune reaction to the pathology.

## Materials and Methods

### Experimental Model

The genotype of the rTg4510 mouse line is 129S6.Cg-Tg(Camk2a-tTA)1Mmay/JlwsJ; Fgf14Tg(tetO-MAPT*P301L)4510Kha/J while formerly 129S6.Cg-Tg(Camk2a-tTA)1Mmay/JlwsJ; FVB-Tg(tetO-MAPT*P301L)#Kha/JlwsJ. These animals were generated by crossing the 4510 responder line, carrying a human MAPTP301L cDNA downstream a tetracycline operon-responsive element (TRE), to an activator line expressing a tetracycline-controlled transactivator (tTA) under control of the CaMKIIα promoter.

### Study design

For this study 13 male mice of the rTg4510 line were included, 10 at the age of 3.5 months and 3 at the age of 4.5 months. The same age animals were randomly assigned to the sham and sonicated groups (five mice per group) while the older mice were utilized in the optimization and validation of the algorithms. Animals were group-housed under standard conditions (12 hr light/dark cycles, 22°C), were provided with a standard rodent chow (3 kcal/g; Harlan Laboratories, Indianapolis, IN, USA) and water ad libitum. All procedures involving animals were approved by the Columbia University Institutional Animal Care and Use Committee.

All mice involved in the study (sham and sonicated) were anesthetized with a mixture of oxygen and 1-2% isoflurane (SurgiVet, Smiths Medical PM, Inc.,WI), placed prone with the head immobilized by a stereotaxic apparatus (David Kopf Instruments, Tujunga, CA) and depilated to expose the suture anatomy and minimize acoustic impedance mismatch. Identification of the parietal and interparietal bone intersection enabled proper positioning of the transducer following a grid-guided targeting procedure 23. Targeting the hippocampal formation involved positioning of the transducer 3 mm anteriorly to the lambdoid suture, and 1.5 mm laterally towards the left hemisphere to cover the dorsal part, while 2 mm anteriorly and 3.2 mm laterally for the ventral part. A bolus of 0.1 μL/g of body mass polydisperse manufactured in-house 45,46 microbubbles diluted in saline (8x10^8^ #/ mL, mean diameter: 1.4 μm) was injected intravenously, in all mice, immediately preceding the sonication.

Five mice (3.5 months old) were sonicated for 60 s, with a pulse length of 6.7 ms and a repetition frequency of 10 Hz at peak negative acoustic pressure (PNP) of 0.45 MPa 17 after accounting for 18% murine skull attenuation. Acoustic emissions were monitored in real time 47 to assess the degree of cavitation. The remaining five mice at 3.5 months of age and the 4.5 months old animals were subjected to the procedure without triggering the transducer (sham groups). All animals underwent magnetic resonance imaging for BBB opening confirmation and safety assessment. The same procedure was repeated once per week for four consecutive weeks while the animals survived for one day after the last treatment prior to sacrifice.

### Focused ultrasound

A single-element, spherical-segment FUS transducer (center frequency: 1.5 MHz, focal depth: 60mm, radius: 30mm; axial full-width half-maximum intensity: 7.5 mm, lateral full-width half-maximum intensity: 1 mm, Imasonic, France), driven by a function generator (Agilent, Palo Alto, CA, USA) through a 50-dB power amplifier (E&I, Rochester, NY, USA) was used to target the hippocampus. A needle hydrophone (HGL-0400, Onda Corp., Sunnyvale, CA) was used for the transducer calibration, which measured the acoustic beam profile in a tank filled with degassed water. A central void of the therapeutic transducer held a pulse-echo ultrasound transducer (center frequency: 10 MHz, focal depth: 60 mm, radius 11.2 mm; Olympus NDT, Waltham, MA) used for alignment, with their two foci aligned. The imaging transducer was driven by a pulser-receiver (Olympus, Waltham, MA, USA) connected to a digitizer (Gage Applied Technologies, Inc., Lachine, QC, Canada). A cone filled with degassed, distilled water was mounted onto the transducer assembly. The transducers were attached to a computer-controlled 3D positioning system (Velmex Inc., Lachine, QC, Canada).

### Magnetic resonance imaging

Following the ultrasound procedure, all animals underwent scanning with the 9.4T MRI system (Bruker Medical, Boston, MA). The mice were placed in a birdcage coil (diameter 35 mm), while being anesthetized with 1 - 2% isoflurane and respiration rate was monitored throughout the imaging sessions. MR images were acquired using a contrast-enhanced T1-weighted 2D FLASH sequence (TR/TE 230/3.3 ms, flip angle: 70°, number of excitations: 6, field of view: 25.6 mm × 25.6 mm, resolution 100 μm x 100 μm x 400 μm), 30 min following the intraperitoneal bolus injection of 0.3 ml gadodiamide (GD-DTPA) (Omniscan^TM^, GE Healthcare, Princeton, NJ). As previously reported, gadodiamide provides spatial information of the BBB opening by temporally enhancing the MR signal relative to the ultrasound parameters 48. In addition, T2-weighted MRI was performed one day after the sonication to detect any potential damage using a 2D RARE sequence (TR/TE 2500/3.3 ms, echo train: 8, number of excitations: 8, field of view: 25.6 mm × 25.6 mm, resolution 100 μm x 100 μm x 400 μm). The sequences employed in this study have been previously optimized by our group 17.

### Immunohistochemistry

All animals were transcardially perfused (30 mL PBS followed by 60 mL 4% paraformaldehyde) one day after the last treatment. The brains were extracted, immersed in PFA fixative for 24 h and then in 30% Sucrose for at least 2 days prior to freezing. After brains were frozen on dry-ice, cryostat-cut coronal hippocampal sections (35 μm) were collected in anti-freezing solution. Then, 2-3 free-floating sections of the dorsal hippocampus with a 6-sections gap were processed for immunohistochemistry. The sections were sequentially washed in 0.1M PBS, treated with 5% donkey serum in 0.3% PBST for thirty minutes with the addition of mouse seroblock for the last ten minutes followed by overnight incubation at 4 °C with the primary antibodies: i. mouse anti-human-phospho-tau (AT8, 1:500, Sigma) and rabbit anti-CD68 (1:500, abcam), ii. mouse anti-human-phospho-tau (AT8, 1:500, Sigma) and rabbit anti-Iba1 (1:500, abcam), iii. mouse anti-β-tubulin III (1:500, Sigma) and rabbit anti-NeuN (1:500, abcam) antibodies. Then, sections were washed in PBS and incubated for 60 min in 0.3% PBST with 5% donkey serum containing the respective donkey-raised secondary antibodies, Texas Red-, Alexa488- and Alexa405-conjugated (1:1000). After the incubation, the sections were washed again in PBS and mounted on slides. The slides were merged into 0.3% sudan black for five minutes, rinsed with 70% ethanol, washed three times with 0.02% Tween20 in PBS and treated with Hoechst 33342 (5 μm/ml) for ten minutes, when cell visualization was necessary. Finally, the slides were washed with PBS and cover slipped with Fluoromount solution.

### Confocal microscopy

The large field confocal images of the hippocampus were captured on a 20x objective of a Nikon confocal microscope (Nikon Instruments Inc., Melville, NY, USA) with the same exposure parameters for the lasers. Tile (mosaic) and a Z-stack (2 μm step, 7 series) were necessary were acquired and processed with ImageJ to produce the resulting maximum intensity image. For colocalization assessment, three single-tile and non-overlapping images covering the CA1 region were captured on a 60x objective of the same microscope. The acquired Z-stack (1 μm step, 13 series) was analyzed with ImageJ to construct the orthogonal views 6 and the corresponding video included in the Supplementary Material. Custom algorithms in MATLAB (R2017a, MathWorks, Inc., Natick, MA, USA) further analyzed all images.

### Quantification Algorithms

#### MR Volumetry

The enhancement in the horizontal plane of MR images was quantified by volumetric analysis encompassing the hippocampal formation 48. Two same-size ellipsoidal regions of interest (ROI) were constructed, covering the targeted the contralateral sides. The sum of the pixels in the ipsilateral ROI surpassing the mean value of the contralateral ROI yielded the area of the BBB opening while performing the same analysis in 17 consecutive slices, matching the dimensions of the focal beam, resulted in the BBB opening volume reported herein 48.

#### Confocal image analysis

**Figure 1:**
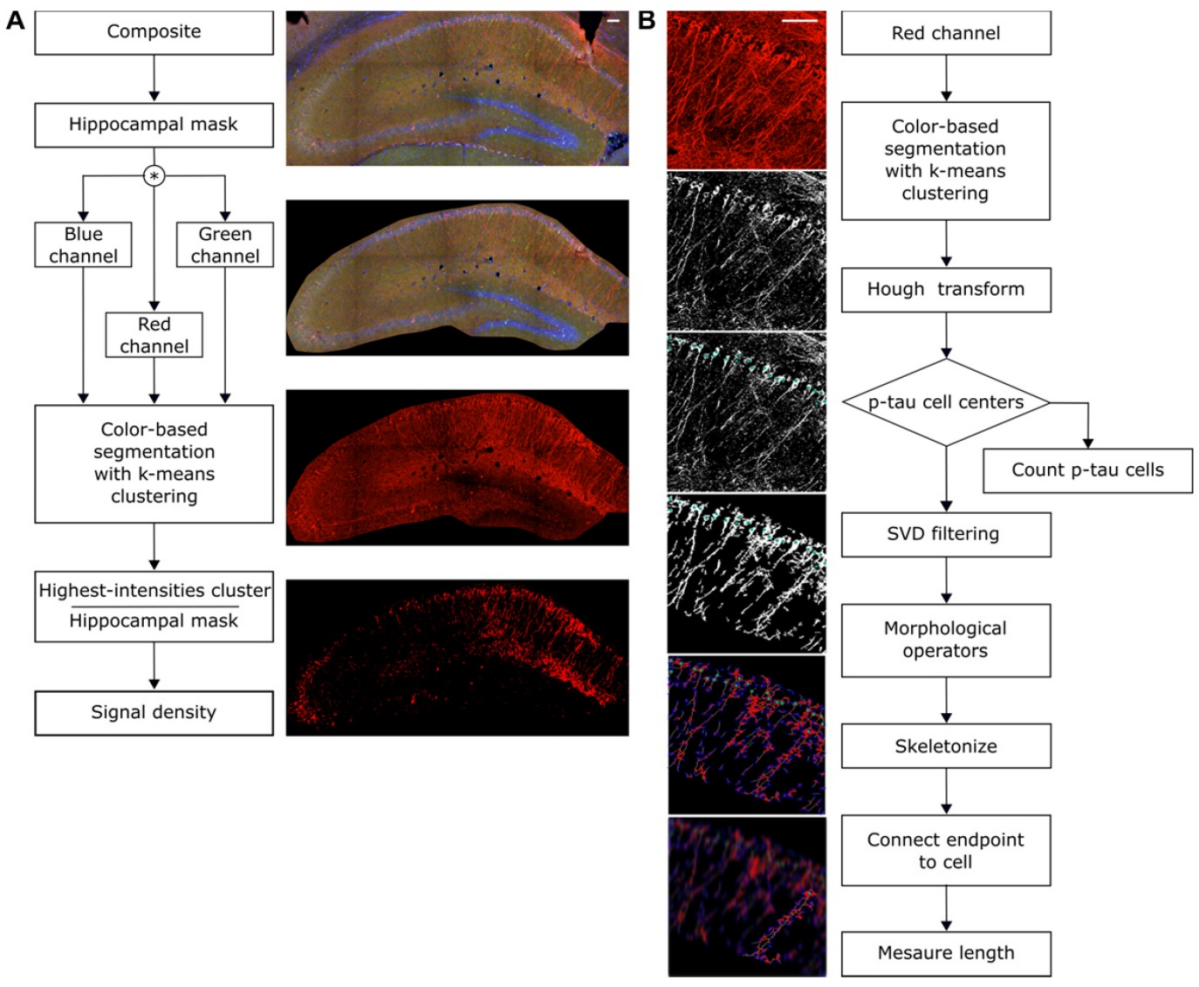
Quantification algorithms. **A:** The structural algorithm performed color-based segmentation with k-means clustering on the red channel filtering out weaker intensities. The Hough transform detected the cells as bright circular objects on a dark background, providing their total count and the corresponding coordinates. Singular value decomposition (SVD) was used to filter the image followed by morphological operators that skeletonized and revealed the spine of the process. The coordinates of each cell were then used to initiate a stemming process search until an endpoint was reached. The distance of the endpoint to the cell center was measured. **B:** The intensity algorithm utilized the composite image as input to generate a hippocampal mask, omitting the irrelevant neighboring structures. The mask was applied to all three channels separately followed by color-based segmentation via k means clustering. For every channel the density is reported as the ratio of the pixels belonging to the cluster with the highest value over the pixels constituting the entire hippocampal mask. Scale bar, 100μm.

The hippocampal formation was isolated from the surrounding brain tissue utilizing the merged-channel images (composite), thus constructing a hippocampal mask. For individual biomarker analysis, the masked composite maximum intensity images were split into the constituent channels. Color-based segmentation by k-means clustering was applied in every channel to determine the sum of pixels belonging to the highest-intensities cluster that was then normalized by the sum of all pixels composing the hippocampal mask (Figure 1A).

Additionally, a structural algorithm was developed to quantify the number of cells and the length of the processes affected by phosphorylated tau (Figure 1B). As previously described, the hippocampal mask was constructed and applied onto the red channel followed by color-based segmentation. The Hough transform was employed to detect circular objects and identify pathological cell centers. Residual noise was eliminated by singular value decomposition. Morphological operators enabling the connection of neighboring pixels and skeletonization, revealed the backbone of the processes along with branches and endpoints. The algorithm utilized the cell center coordinates as the initial point to search for the presence of a skeleton in a defined neighborhood. Every path was followed through all potential branches until reaching an endpoint. The distance of that endpoint to the cell center was measured and the longest 5% of the paths leading to the same cell were measured and averaged. Since the hypothesis of this study was that focused-ultrasound induced BBB opening reduces phosphorylated tau especially from the CA1 processes, false positives (erroneously finding a long path other than the true path) were preferred as errors avoiding favorable bias.

To report the difference in pyramidal processes length between groups, a Monte Carlo simulation was performed. The simulation constructs the probability density function (PDF) for the processes length of each hemisphere (sham contra, sham ipsi, sonic contra and sonic ipsi) followed by random sampling from each PDF and pairwise subtraction of the random samples 49. The subtraction is demonstrated as the cumulative density distribution revealing the percent of length difference.

Both the intensity- and structural- algorithms as well as the Monte Carlo simulation were evaluated in terms of accuracy by comparing the pathology of rTg4510 mice at different ages. As the disease progresses, the cells affected by the pathology should significantly increase as phosphorylated tau spreads from the processes that become shorter 2,50. Pathological deterioration is also reflected by increased activity of immune cells (microglia and/or macrophages) evident by elevated CD68-positivity 51. Increased cell population and CD68-positivity accompanied by decreased length of the processes affected by phosphorylated tau was indeed detected in the older mice with the associated changes in intensity (Figures S1 and S2).

To estimate the statistical dependence between p-tau and microglia, and report on their colocalization probability, standard methods were employed; i. the Pearson's correlation coefficient (PCC) and ii. Manders' overlap coefficients; M1 and M2, where the threshold was set to the estimated background mean value 52.


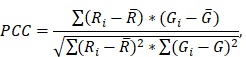



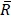
 and 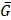 are the mean red and green channel values.


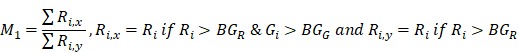



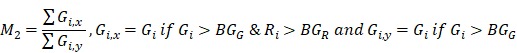


where *R_i_* and *G_i_* are the pixel intensities of the red and green channel respectively while *BG_R_* and *BG_G_* are the mean background values of the red and green channel respectively.

The equations describing the coefficients were implemented in MATLAB. Following the isolation of the CA1 sector from the surrounding tissue, the dependency coefficients were calculated using the raw intensities as inputs. Pixels outside the region of interest were assigned the arithmetic representation for Not-a-Number (NaN) that does not affect the calculation of the coefficients.

### Statistical analysis

All values are expressed as means ± standard error of the mean (SEM). Differences between the means of the sham and treated groups as well as the ipsilateral and contralateral to the sonication sides were analyzed using 2-way repeated measures ANOVA. Statistical analysis was performed on the mean value obtained per animal; however, all samples (2-3 slices per mouse brain) are presented in the bar plots included in the study. Longitudinal analysis of the BBB opening volume and differences in the means distributed over the hippocampal subfields were analyzed with multiple Student's t tests. The P values in both analyses were adjusted based on the Holm Sidak post hoc correction. All statistical analyses were performed using Prism 8 (Graphpad Software, San Diego, CA, USA) and the null hypothesis was rejected at the 0.05 level. Throughout the manuscript the “F” value in respect to the associated degrees of freedom is provided with the P value adjusted to the corrected multiple t-tests. The significance levels correspond to: * P<=0.05, ** P<=0.01, *** P<=0.0005, **** P<0.0001.

## Results

### FUS-induced BBB opening did not differ in volume between groups and across weeks

**Figure 2:**
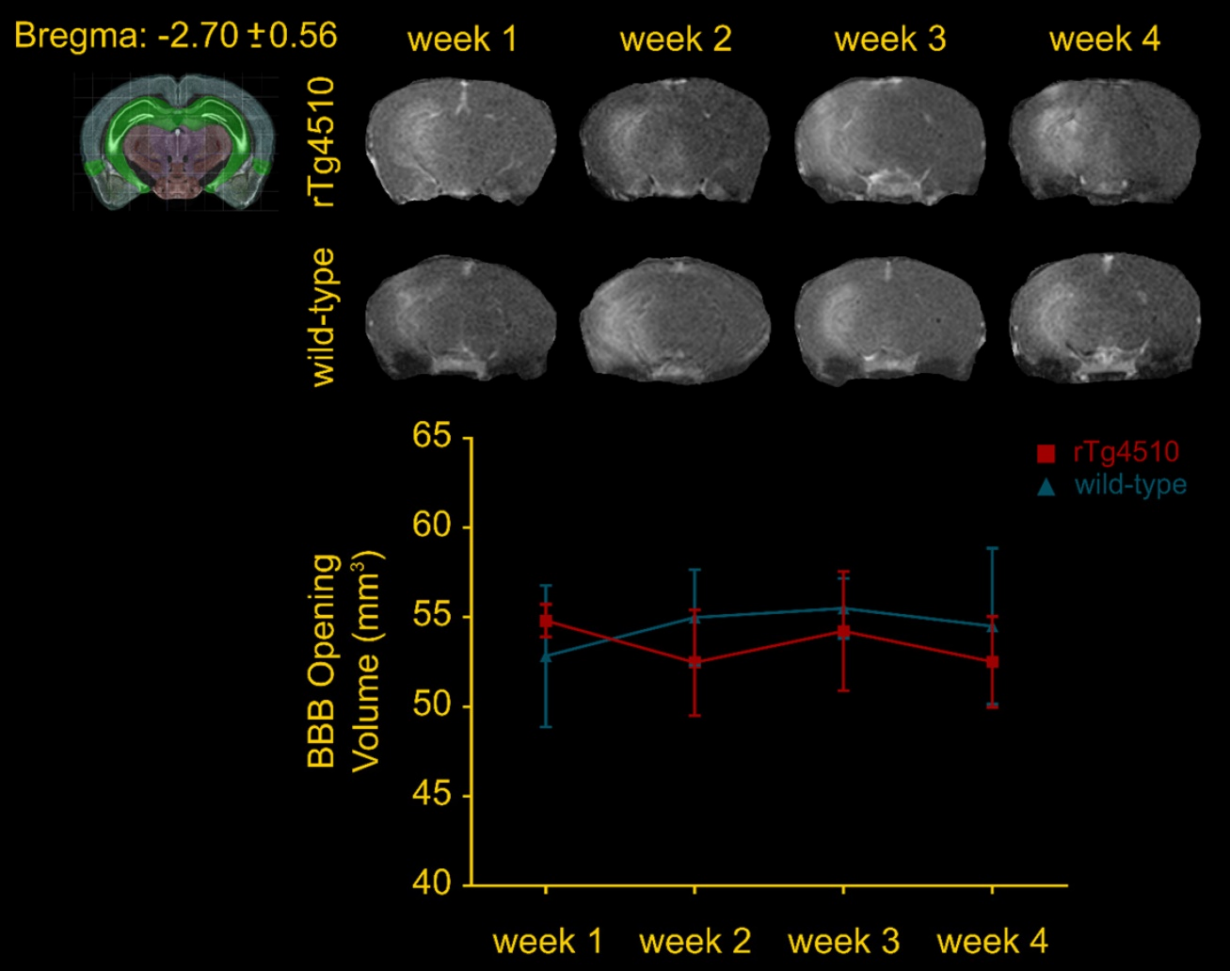
Volumetric analysis of the blood-brain barrier opening from the contrast-enhanced T1-weighted MR coronal images. The volumes of the transgenic animals were at the order of 54.8 ± 2.02 mm^3^, 52.46 ± 6.59 mm^3^, 54.22 ± 7.44 mm^3^ and 52.5 ± 5.64 mm^3^, while 52,83 ± 8.81 mm^3^, 54.98 ± 5.97 mm^3^, 55.48 ± 3.75 mm^3^and 54.49 ± 9.76 mm^3^ of the wild-type mice for the four consecutive weeks. Longitudinal analysis did not show any significant difference across weeks. Additionally, the opening volumes did not differ between transgenic and wild-type animals within the same week interval.

Confirmation of the BBB opening and volume assessment was performed prior to the immunohistochemical analysis to evaluate potential differences in the opening volume. Representative coronal planes of the BBB opening at Bregma -2.70 ± 0.56 are shown in Figure 2 for each group per week of sonication. Quantification of the opening volume (transgenic; wild-type, week 1: 54.8 ± 0.9 mm^3^; 52.83 ± 3.9 mm^3^, week 2: 52.46 ± 2.9 mm^3^; 54.98 ± 2.6 mm^3^, week 3: 54.22 ± 3.3 mm^3^; 55.48 ± 1.6 mm^3^, week 4: 52.5 ± 2.5 mm^3^; 54.49 ± 4.3 mm^3^) did not reveal any significant differences in the opening size across weeks suggesting that repeated ultrasound application did not compromise the integrity of the barrier at least at this age (3.5-4.5 months old). Moreover, the comparable BBB opening volumes of transgenic to wild-type animals indicated a similar response of the BBB to the ultrasound without evidence of edematous incidences as indicated by the T2-weighted images (Figure S3).

### FUS-induced BBB opening does not compromise neuronal integrity

**Figure 3:**
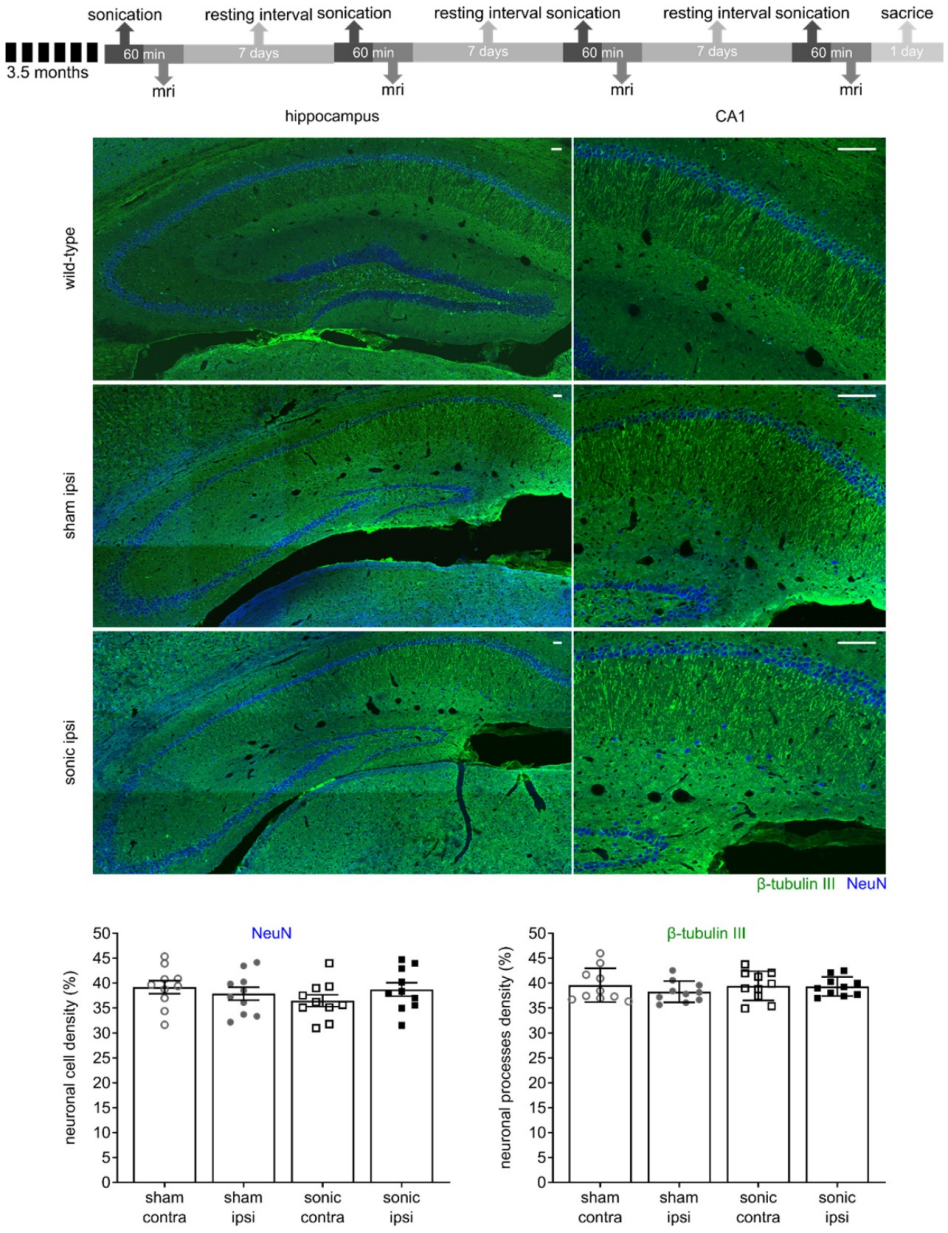
The timeline of the experimental procedure is shown at the top. Briefly, five mice of the rTg4510 line at 3.5 months of age were recruited for four sonications, once per week and were sacrificed a day after the last treatment. MRI was performed after each sonication to confirm targeting accuracy and successful opening. Two slices per transgenic brain were counterstained for neuronal cells with the anti-NeuN and anti-β-tubulin ΙΙΙ antibodies along with brain slices from a wild-type mouse to assess neuronal integrity. Neuronal compromise could be qualitatively observed in transgenic animals compared to healthy mice by the decrease in the NeuN signal and the non-uniform signal emitted by the neuronal processes. However, no significant differences emerged from the application of ultrasound as shown by the quantitative measures. The mean (± standard deviation) neuronal cell density was 39.18 ± 4.25% and 36.46 ± 3.77%, for the sham contralateral and ipsilateral side while 37.87 ± 4.14% and 39.18 ± 4.09% for the corresponding sides of the sonicated brains. Respectively, the mean value (± standard deviation) for the neuronal processes density was 39.31 ± 1.91% and 39.43 ± 2.93% for the sham contralateral and ipsilateral side while 38.25 ± 2.13% and 39.56 ± 3.3% for the corresponding sides of the sonicated brains. Scale bar, 100 μm.

FUS applied within the safety regime has been proven beneficial 26,35,53 while vascular incidences have been reported in sonication protocols involving overlapping regions and repeated applications 43. Despite operating in a safe ultrasound parametric space evaluated in previous studies 33,53, we were interested in determining any potential effects of the technique on the neuronal integrity. Therefore, two slices per transgenic brain were counterstained for neuronal cells with the anti-NeuN (neuron-specific nuclear protein) and anti-β-tubulin ΙΙΙ antibodies (neuron-specific microtubule element) along with brain slices from a wild-type mouse to assess the sensitivity of the biomarkers. Neuronal compromise was qualitatively evident when comparing the transgenic to the wild-type brains by the dentate gyrus' shrinking due to the neuronal depletion in the rTg4510 mouse model at this age (Figure 3) 50. Additionally, the neuronal processes in the wild-type hemispheres and particularly the CA1 sector were positive to the anti-β-tubulin III antibody along their in-plane length. On the contrary, the processes in the transgenic animals appeared more fragmented. Despite the differences observed between transgenic and wild-type brains though, no significant differences emerged from the application of ultrasound. Quantification of the two channels separately based on their intensity normalized by their background (See Materials and Methods and Figure 1A) showed that neither neuronal cells nor processes differed between the sonicated and sham brains (Figure 3). The neuronal cell density was 39.18 ± 1.41% and 36.46 ± 1.25%, for the sham contralateral and ipsilateral side while 37.87 ± 1.3% and 39.18 ± 1.29% for the corresponding sides of the sonicated brains. Respectively, the neuronal processes density was 39.31 ± 0.63% and 39.43 ± 0.97% for the sham contralateral and ipsilateral side while 38.25 ± 0.67% and 39.56 ± 1.04% for the corresponding sides of the sonicated brains. These observations suggest that the ultrasound parameters applied in this study did not negatively impact the neuronal integrity at least to the sensitivity level of our detection.

### FUS-induced BBB opening reduces phosphorylated tau (p-tau) from the hippocampus

**Figure 4:**
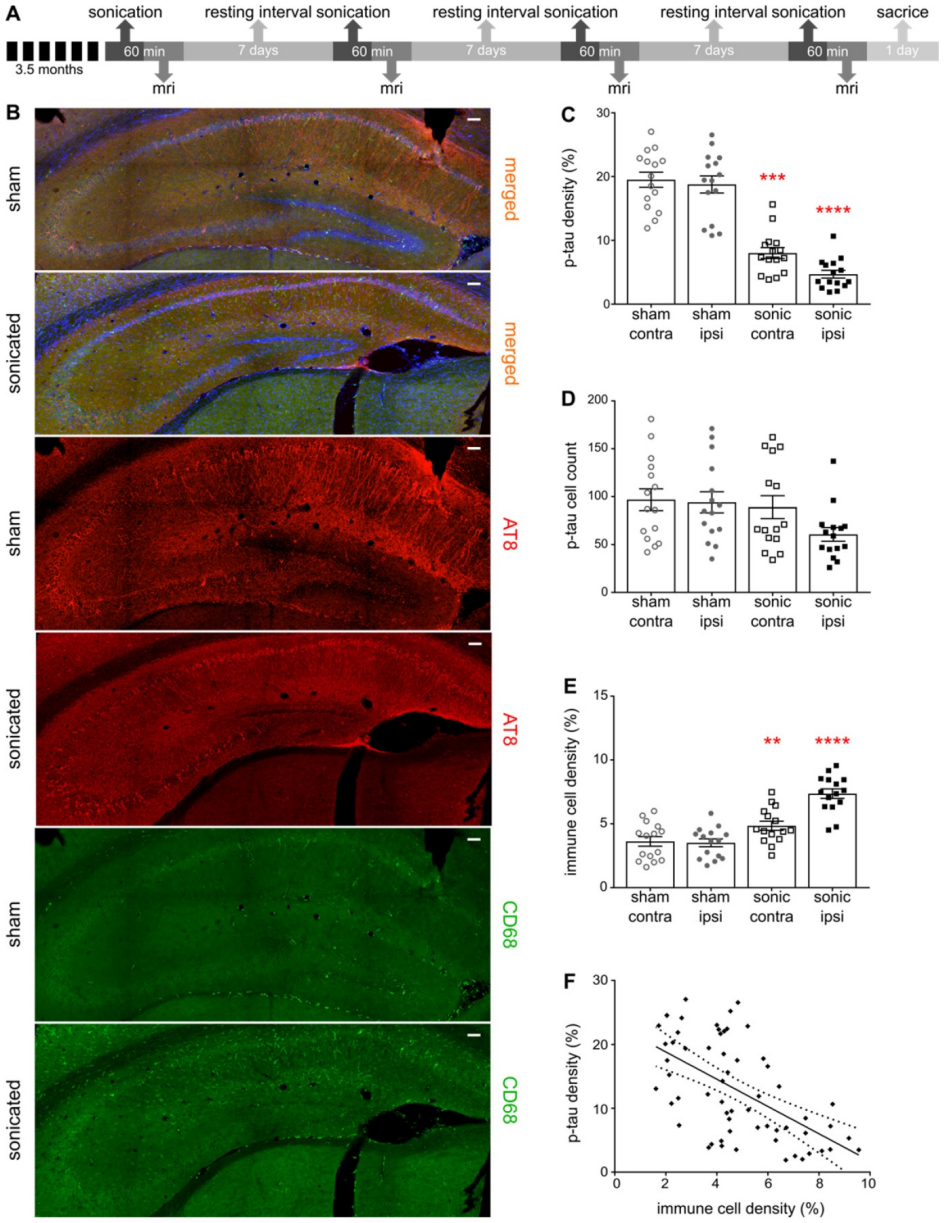
** A:** The timeline of the experimental procedure. For this immunohistochemical analysis brain slices from the transgenic animals were counterstained for phosphorylated tau with the AT8 antibody and immune cell activation with the CD68 antibody while imaged by confocal microscopy. **B:** Representative composite images of the sham and sonicated ipsilateral hemispheres are shown with the red channel corresponding to the signal emitted from phosphorylated tau, the green channel reflecting the immune response and the blue channel representing the cell-dye Hoechst 33342. **C:** Bar scatter plot representation of the samples showing a significant reduction in the p-tau signal when comparing the hemispheres of the sham and the sonicated brain. In particular, we observed a reduction in the p-tau signal at the order of 57.35% (F_1,8_ = 34.32;P=0.0004) when comparing the contralateral hemispheres of the sham and the sonicated brains, while 72.65% (F_1,8_ = 34.32;P<0.0001) when comparing the ipsilateral hemispheres. **D:** On the other hand, the total cell numbers detected by the algorithm did not differ among the groups or the hemispheres **E:** Immune cell activation was confirmed by the signal obtained from the CD68 marker. Immune system upregulation was expected due to pathology but a 54.41% increase (F_1,8_ = 46.4;P<0.0001) was observed in the hemisphere treated with ultrasound while a 41.6% (F_1,8_ = 46.4;P=0.0064) increase in its contralateral side compared to the control brains. **F:** Regression analysis between the p-tau signal and the immune cell activation yielded a significant deviation of the slope from the zero value suggesting a correlation between the two parameters (r^2^=0.3285; β=-2.136; P<0.0001). Scale bar, 100 μm.

Establishing the neuronal integrity, we were then interested to investigate the response of phosphorylated tau protein to the application of ultrasound and the BBB opening. Frozen floating tissue was processed for immunohistochemistry by incubation with antibodies against phosphorylated tau and immune cell reactivity using AT8 (a monoclonal antibody recognizing AD-related phosphorylation at Ser202/Thr205 on human tau protein) and CD68 (monoclonal antibody binding to the corresponding lysosomal receptor of macrophages) respectively. Confocal microscopy enabled visualization of the p-tau spatial profile as well as correlation with the immune response. The distribution of phosphorylated tau changes with disease stage with p-tau being present in the processes at early stages, but more pronounced in the somatodendritic compartment as the disease worsens 2. Quantification of p-tau signal-intensity showed a significant reduction in the hemisphere treated with ultrasound (Figure 4B-C). In particular, we observed a reduction in the p-tau signal to the order of 57.35% (F^1^,^8^ = 34.32;P=0.0004) when comparing the contralateral hemispheres of the sham and the sonicated brains, and 72.65% (F^1^,^8^ = 34.32;P<0.0001) when comparing the ipsilateral hemispheres (Figure 4C). However, the total p-tau cell numbers detected by the algorithm did not differ among the groups or the hemispheres as shown in Figure 4D.

### FUS-induced BBB opening induces an immune response that correlates with p-tau reduction

BBB opening induced by FUS has been shown to trigger an immune response determined by the energy deposited and the selected ultrasound parameters 25. Under the current sonication regime, microglia activation was expected as the brain-residing myeloid cells reach for the sites of BBB leakage 25,26. Microglia are inherently activated by the pathology 51, however, the binding ligand that induces the conformational changes governs their performance in the brain 41,42. Additionally, brain trafficking of peripheral immune cells has been associated with mitigation of neurological diseases attributed to their strong phagocytic capacitance 54. However, peripheral immune cells are restrained from entering the brain, yet FUS has been shown to facilitate their infiltration by transiently disrupting the BBB 25,43,44. In this study, significant activation of immune cells at the order of 54.41% (F^1^,^8^ = 46.4;P<0.0001) and 41.6% (F^1^,^8^ = 46.4;P=0.0064) was observed in the hemispheres treated with ultrasound and its contralateral side compared to the untreated brains (Figure 4E). Moreover, regression analysis between immune cells and p-tau signal revealed a negative correlation showing increased immune response in the brains with reduced phosphorylated tau presence (Figure 4F). Despite the relatively low correlation coefficient (r^2^=0.3285), the slope of the regression significantly deviated from zero (β=-2.136; P<0.0001) indicating that the two variables vary together in the opposite direction.

### FUS-induced BBB opening reduces phosphorylated tau (p-tau) from the CA1 pyramidal neuronal processes

**Figure 5:**
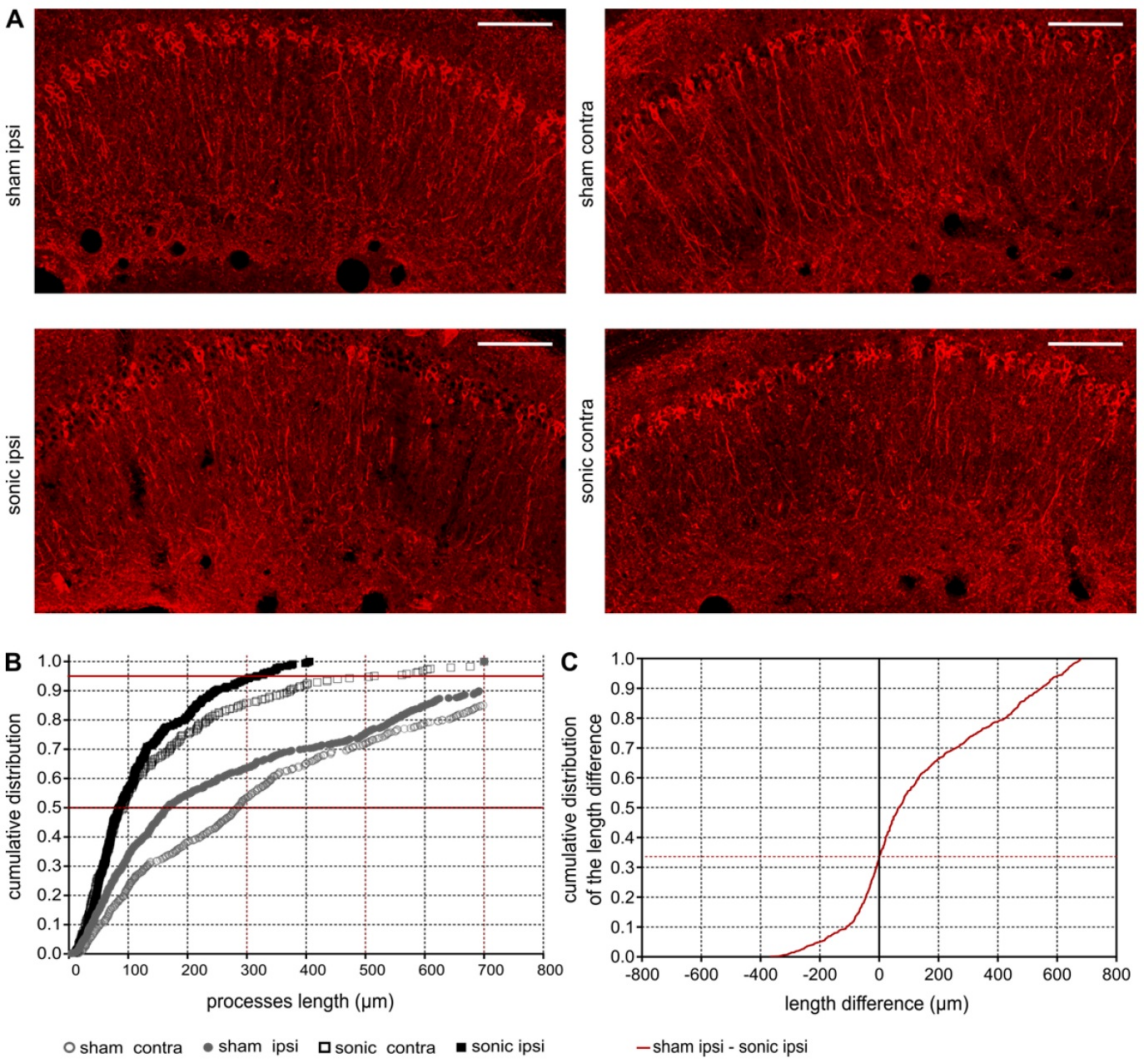
** A:** Phosphorylated tau signal emitted from the affected pyramidal neurons of the ipsilateral and contralateral side in the CA1 sector of the sham and sonicated brains. **B:** Comparison of the cumulative density function (CDF) of the p-tau processes length as obtained from each group (sham contra, sham ipsi, sonic contra and sonic ipsi). This graph describes the probability (y-axis) of finding p-tau processes of a certain or smaller length (x-axis). The 95^th^ percentile (upper red line) crosses the CDF of the sonicated ipsilateral side at 300 μm (dotted red line), while at 700 μm (dotted red line) for the untreated brain. This finding suggests that the probability of finding a p-tau neuronal process equal or smaller than 300 μm in the sonicated brain and 700 μm in the unsonicated brain is 0.95. **C:** The cumulative density function of the difference in p-tau processes length between the sham and sonicated hemisphere (ipsi sham-ipsi sonic). The zero crossing denote the probability of p-tau processes having the same length between the two hemispheres. From this CDF it can be observed that 68% of the neurons on the sham ipsilateral side are longer than those on the sonicated ipsilateral side. Scale bar, 100 μm.

Upon observation of the reduction in phosphorylated tau signal emitted from the sonicated hippocampus, further investigation of its spatial distribution along the pyramidal neuron was necessary. Given the CA1 being the hippocampal sector most affected by the pathology at this age in the rTg4510 model 50 (Figure S4) further quantification of the processes length affected by p-tau followed. In Figure 5A, it is qualitatively shown by the red channel that p-tau affects the neuronal processes along their in-plane length (longer p-tau processes) while application of ultrasound decreases the neuronal processes depletion (shorter p-tau processes). Quantification of the p-tau processes length was performed by a custom algorithm that beginning from the cell soma tracked the pathological marker along the axon and returned its length (See Materials and Methods and Figure 1B). In Figure 5B the cumulative density function (CDF) demonstrates the p-tau processes length across the four groups (sham contra, sham ipsi, sonic contra and sonic ipsi). The 95^th^ percentile, denoted by the upper red line, crosses the CDF of the sonicated ipsilateral side at 300 μm (dotted red line), while at 700 μm (dotted red line) for the untreated brain. This finding suggests that the probability of finding a p-tau neuronal process equal or smaller than 300 μm in the sonicated brain and 700 μm in the unsonicated brain is 0.95. Despite establishing the high probability of finding shorter p-tau processes in the sonicated brain, to report on the differences in the p-tau processes length between the groups, a Monte Carlo simulation was designed 49. The probability density function of the p-tau processes was constructed for the four groups. Samples from the distributions were randomly drawn, subtracted from each other pairwise and the cumulative density function of the resulting differences in length is reported herein 55,56. The CDF of the length difference in p-tau processes between the sham and sonicated hemispheres is shown in Figure 5C. The probability of the p-tau processes having the same length is 0.32 (zero crossing) suggesting that 68% of the p-tau processes in the untreated brains are longer than the sonicated ones. Another observation that could be extracted from this figure is that the probability of a p-tau process in the untreated brain being up to 600μm longer than the sonicated brain is 0.94. Similar observations can be respectively drawn for the remaining groups in Figure S5. The accuracy of this method in the current application was evaluated by performing the equivalent analysis in the different rTg4510 age groups which followed the expected pathological outcomes (Figure S2).

### Microglia colocalization with phosphorylated tau in the CA1 sector

**Figure 6:**
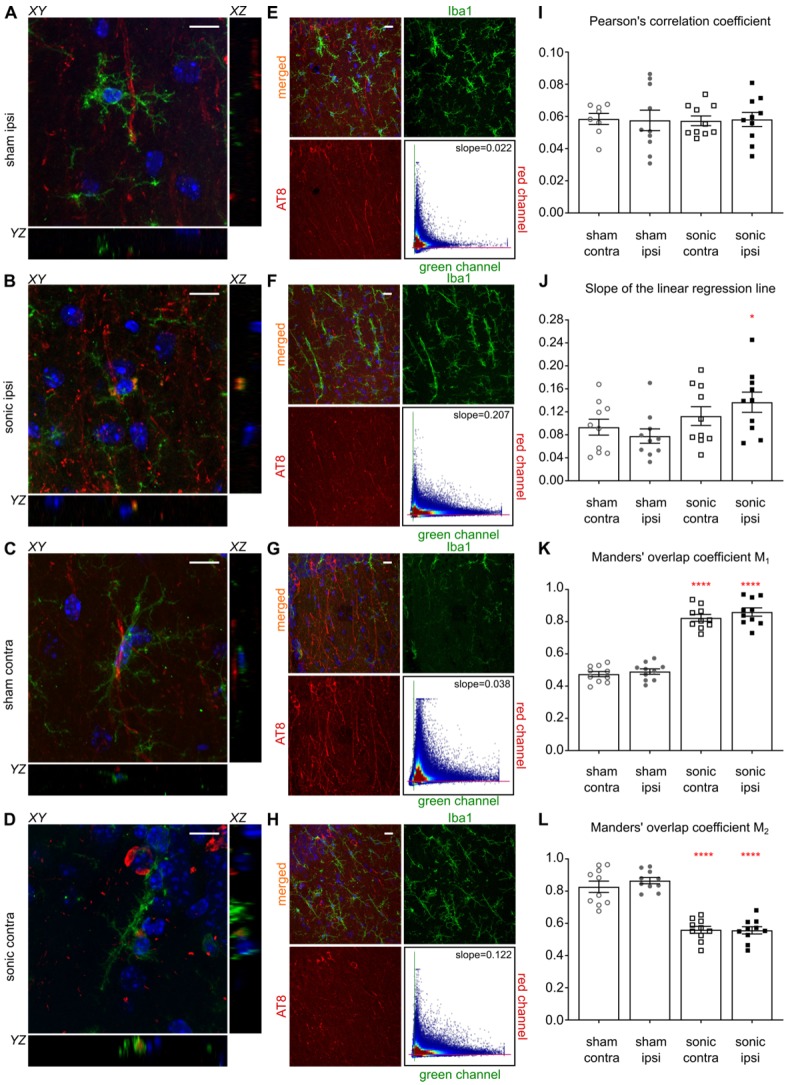
Microglia colocalization with phosphorylated tau protein. For this immunohistochemical analysis, two brain slices from the transgenic animals were counterstained for phosphorylated tau and microglia presence with the AT8 (red) and the Iba1 (green) antibody. Three Z-stack series were captured on a 60x objective covering the CA1 region. **A-D:** Qualitative evaluation of the biomarker colocalization. The three planes, XY (main), XZ and YZ (orthogonal) are presented for a microglia cell. The images are magnified versions of the 60x objective and the corresponding video included in the Supplementary Material. **A, C:** The microglia are shown to verge on the phosphorylated tau but not engulf it as indicated by the lack of the channel overlap in all three planes. **B, D:** The microglia seem to engulf fragments of phosphorylated tau after the application of ultrasound in both hemispheres of the sonicated brains. The overlap of the two channels is consistent in all three planes. **E-H:** The four panels follow the same structure: a composite image showing the merged and monochromatic images of the isolated CA1 sector and the scatterplot of the two channels. The scatterplot colormap indicates the pixels density. **E:** The neuronal processes are largely affected by phosphorylated tau while the presence of microglia is evident in the ipsilateral sham hemisphere. The scatterplot has a slope (the slope obtained by performing linear regression between the two channels) of 0.022 suggesting that more pathological tau is present (higher red channel values) than microglia (smaller green channel values) while Pearson's correlation coefficient (PCC) is 0.0345 suggesting minor statistical dependence between the biomarkers. **F:** The sonicated hemisphere experienced a reduction in phosphorylated tau and an increase in microglia presence indicated by the higher value of the fitted slope equal to 0.207, while the PCC increased for this case to 0.068. **G:** Similar findings to the ipsilateral hemisphere can be extracted from the contralateral sides where the slope is 0.038 and the PCC of 0.058 in the sham brain while **H:** the slope reached 0.122 and the PCC 0.0.068 in the sonicated brain. **I-L:** Cumulative results on the dependency metrics. Comparable PCC values suggest that ultrasound did not affect the covariance of the two biomarkers. However, a significant increase in the slope of the linear regression line by 43% can be observed between the ipsilateral hemispheres (F[1.6]=6.214;P=0.047). Furthermore, the percent of red-to-green channel contribution, measured here my Manders' M_1_ overlap coefficient, increased significantly by 43% (F[1.6]=162.5;P<0.0001) while the M_2_ coefficient decreased significantly by 36% (F[1.6]=258.9;P<0.0001) suggesting greater overlap of the green with the red channel in the ipsilateral hemisphere. Similarly, M_1_ overlap coefficient increased by 42.6% and M_2_ coefficient decreased by 31.7% in the contralateral hemispheres. Scale bar, 10 μm.

Having established the reduction in p-tau signal in the CA1 sector as well as the increased activity of the immune cells triggered by the application of ultrasound, we were interested in investigating the colocalization probability of the brain-resident macrophages with the phosphorylated tau protein. Existing evidence suggests that the immune response triggered by the FUS-induced BBB opening results in the increased microglia activity 26,31. Therefore, we performed an additional immunohistochemical analysis utilizing the anti-Iba1-antibody (calcium-binding polyclonal antibody) and the anti-phospho-tau antibody described previously. Since channel overlap in one plane does not suffice to justify biomarker colocalization, we investigated the orthogonal views 6 of all microglia cells captured within the images and representative magnified examples (60x objective magnified by a factor of three) are shown in Figures 6A-D. Qualitative evaluation of the channel overlap revealed that microglia verge on the p-tau processes and follow morphologically their structure in the sham brains without, though, engulfing them, as shown in Figures 6A,C. However, following the application of ultrasound, fragments of p-tau can be found within the microglia in both hemispheres of the sonicated brains (Figure 6B,D) as suggested by the channel overlap in all three planes (the video is included in the Supplementary Material). Quantitatively, Figures 6E-H include representative composite images from the CA1 region of each group with the corresponding monochromatic channels, where the green corresponds to the Iba1 biomarker and the red to the p-tau pathology. Each scatterplot shows the distribution of the pixels in the green-red coordinate system providing information on the covariance of the markers. The scatterplot colormap indicates the pixel density. Figures 6E and 6G demonstrate the spatial distribution of the microglia and p-tau pathology in the sham brain with a PCC of 0.0345 for the ipsilateral side and 0.0495 for the contralateral side. The slope of the linear regression line between the two channels is 0.0273 for the ipsilateral and 0.0386 for the contralateral sham hemisphere. These findings suggest a positive covariance of the two channels while the small value of the slope suggests that for high red channel intensities the green channel values remain small.

In Figures 6F and 6H, representative examples of the sonicated brain are shown with a PCC of 0.068 for the ipsilateral and 0.0583 for the contralateral hemisphere with the corresponding slopes on the order of 0.207 and 0.122 respectively. The increase in the slope could be attributed to the reduced signal intensity in the red channel accompanied by the increase in green channel intensity while the PCC values were found comparable between groups. As particularly shown in the Figures 6F and 6H scatterplots, the majority of the pixels spreads towards the higher values of the green channel while maintaining a relatively low value in the red channel. This finding suggests a decrease in the signal emitted from phosphorylated tau accompanied by an increase in the immune response as previously shown in Figure 4E. Cumulative results from all animals show comparable PCC values (Figure 6I) accompanied by a significant increase in the scatterplot slope on the order of 43% (F[1.6]=6.214;P=0.047) for the ipsilateral hemispheres along with a 26.7% increase, albeit not significant, for the contralateral hemispheres after the application of ultrasound (Figure 6J). Furthermore, the percent of red-to-green channel contribution, measured here by Manders' M1 overlap coefficient, significantly increased by 43% (F[1.6]=162.5;P<0.0001) for the ipsilateral hemisphere following FUS while the M2 coefficient decreased significantly (F[1.6]=258.9;P<0.0001) by 36.0% suggesting greater overlap between the green and the red channel. Similar findings were observed on the contralateral hemispheres with a 42.6% increase in the M1 coefficient and a 31.7% decrease in the M2 coefficient. The changes in the Manders' coefficients are summarized in Figures 6K and 6L.

## Discussion

FUS coupled with the administration of microbubbles has been proposed as the only non-invasive technique to transiently, locally and reversibly open the BBB. Although repeated ultrasound application has not been found to alter the BBB response longitudinally 18,57, little is known regarding the BBB opening volume in transgenic models and wild-type or aged animals 16. Longitudinal quantification of the BBB opening volume revealed comparable outcomes while comparison of the groups did not show any differences in the opening volumes (Figure 2). Furthermore, the acquisition of safety scans indicative of edema occurrence provided negative results confirming the tolerability of the technique (Figure S3). Along the same lines, microscopic investigation of the ultrasound impact on the neurons was performed suggesting that neuronal cells were not compromised (Figure 3).

Alzheimer's disease is a chronic neurodegenerative disorder characterized by the accumulation of amyloid plaques and neurofibrillary tangles in the brain 1. FUS has emerged as a potential modulatory stimulus driving a contained but simultaneously beneficial immune response that has been proven to alleviate amyloidosis 26,35. Herein we show that the complementary hallmark of the pathology, phosphorylated human tau protein, could be also mitigated by the ultrasound-triggered upregulation of the immune system. In addition to the overall reduction in p-tau from the hippocampal formation, the spatial distribution of the pathological marker, primarily in the CA1 region, changed after the application of ultrasound (Figures 4-6). In particular, smaller segments of the neuronal processes were affected by p-tau without an associated increase in p-tau affected cell somas (Figure 4B-E). Monte Carlo simulations confirmed the reduction in phosphorylated tau from the pyramidal processes as their length decreased significantly with the application of ultrasound (Figure 5). The CA1 pyramidal neurons were found to be primarily affected by the pathology (Figure S4) 50 but were also significantly benefitted from the treatment since the processes affected by p-tau were shorter in length. Overall, ultrasound was considered to have attenuated the pathological progression that is associated with a significant increase of p-tau in neuronal cells (Figure S1). These findings are in agreement with the study of Nisbet et al. 28 that reported on the decrease of phosphorylated human tau protein in the pR5 line. Both studies investigated the tau levels expressed by the mutant human transgene. On the other hand, Kovacs et al. 58 measured the inherent mouse tau levels in wild-type mice reporting an increase in phosphorylated tau after application of repeated FUS. Therefore, further investigation of the differences in brain response to ultrasound in physiological and pathological conditions is necessary.

The link between microgliosis and tau pathology is still debated in the existent literature 39,40. Stimulus-dependent conformational changes and dystrophy have been associated with tau pathology while re-programming of the microglia to healthier phenotypes has recently emerged as a therapeutic strategy 41,42. The beneficial effects of microglia on Aβ pathology have been supported by studies showing clearance of the plaques after FUS-induced BBB opening 26,27 and this seems to be the same for tau pathology as well 28. Moreover, infiltration of peripheral macrophages has been emerged as a potential candidate for the amelioration of Alzheimer's disease 54,59. However, the primary challenge in those studies remains the limited brain infiltration due to the barrier, an obstacle that FUS has been shown to overcome 44. Distinction between microglia and peripheral immune cells including macrophages and monocytes was not attempted in the present study given that the employed antibodies (CD68 and Iba1) are not adequate markers of unique microglia signature 51,60. Therefore, the immune response observed in Figure 4 could be attributed to their synergistic action. The increase in immune cell activation was also accompanied by a reduction in the phosphorylated tau protein in the brains examined (Figure 4E-F). These findings were confirmed by the significant increase in the fitted slope (Figure 6E-I) suggesting a decrease in phosphorylated tau (red channel intensity) and an increase in microgliosis (green channel intensity). In addition, similar observations could be drawn from analyzing Manders' overlap coefficients, since they describe the fraction of one channel in compartments of the second channel 61. Along these lines, significantly increased M1 and decreased M2 indicate that the majority of the phosphorylated tau protein could be found in compartments associated with microglia following the application of ultrasound (Figure 6K,L). Although this finding could also be explained by the reduction in p-tau accompanied by the upregulated immune response (Figure 4E), single-cell, three-dimensional qualitative evaluation of the channel overlap (video is included in the Supplementary Material) strengthens the argument that microglia could engulf the pathological tau (Figure 6A-D) following sonication. Whether FUS-induced BBB opening drives a “healthy” activation of microglia, or infiltrating immune cells 39 help reduce p-tau remains unclear but these mechanisms could explain the positive effect established herein.

While reporting on the previous findings, an interesting observation emerged regarding the bilateral reduction in phosphorylated tau resulting from the unilateral induction of BBB opening (Figure 5A). The effect was significant in both hemispheres compared to the sham brains (Figure 5B). The structural quantification showed that the contralateral neuronal processes were only 150μm longer than the ipsilateral in the sonicated brain at the 95^th^ percentile (Figure 5B). The significant presence of immune cells and their colocalization with p-tau protein in the contralateral-to-ultrasound hemisphere could be driven by the migration of the resident microglia from the sonicated hemisphere to the contralateral side through the integrating tract 62 and/or the infiltration of peripheral cells 25,43,44. A recent study investigating the amyloid plaque load after unilateral sonication in naturally aged canines did not show a significant difference between the hemispheres. Unfortunately, the study was lacking a control pathological brain and therefore the possibility of a bilateral effect could not be investigated 63.

This study demonstrates the initial findings in bilateral reduction of phosphorylated tau in response to unilateral sonication due to the upregulation of the immune response in the rTg4510 mouse model without accompanied compromise of the neuronal integrity, findings that are in agreement with recent observations from alternative tau models 28. The p-tau reduction could suggest deceleration of p-tau spreading and therefore disease progression but additional studies including analytical techniques and behavioral measures are needed to further support this implication.

## Supplementary Material

Supplementary figures.

Supplementary video.

## Abbreviations

AD: Alzheimer's disease
Aβ: amyloid beta
BBB: blood-brain barrier
FUS: focused ultrasound
rTg4510: the mouse strain described in the methods section
PCC: Pearson's correlation coefficient
PNP: peak negative pressure
PRF: pulse repetition frequency
ROI: region of interest
SVD: singular value decomposition
TRE: tetracycline operon-responsive element
tTa: tetracycline-controlled transactivator
