# Supplementary material for: Unilateral Focused Ultrasound-Induced Blood-Brain Barrier Opening Reduces Phosphorylated Tau from The rTg4510 Mouse Model
Source: Theranostics. 2019 Jul 13;9(18):5396–411. doi: 10.7150/thno.28717 (PMC6691580; doi:10.7150/thno.28717)
Supplement: Supplementary file 1 — Supplementary figures. [file thnov09p5396s1.pdf]

**Supplementary Figures**

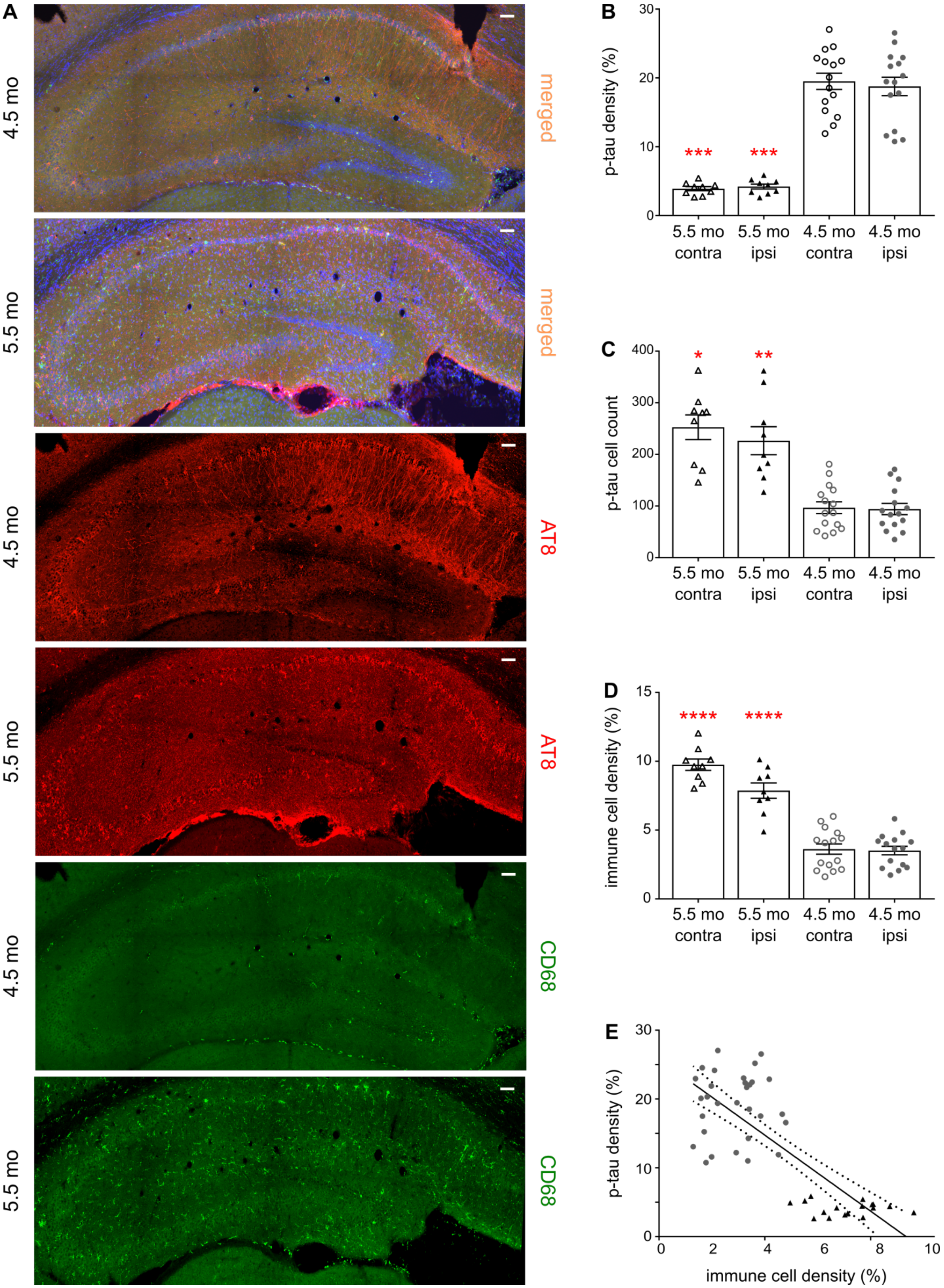

**Figure S1:** Validation of the intensity quantification algorithm by detection of the pathology progression. **A-C:** Tau propagation with age. In the younger sham group (4.5 mo) p-tau can be primarily found in the axonal compartment while in the older sham group (5.5 mo) in the somatodendritic compartment. The pathology progression was confirmed by the 80% ($F_{[1,6]}$=33.83;P<0.0001) decrease in phosphorylated tau accompanied by a 59% ($F_{[1,6]}$=17.83;P=0.0055) increase in affected cells. **D-E:** The immune response was found significantly upregulated (more than twofold) with the progression of the disease as shown by the intensity quantification ($F_{[1,6]}$=121.3;P<0.0001). Additionally, increased immune cell activity correlated well with decreased axonal tau ($r^2$=0.62; β=-2.2; P<0.0001). Scale bar, 100µm.

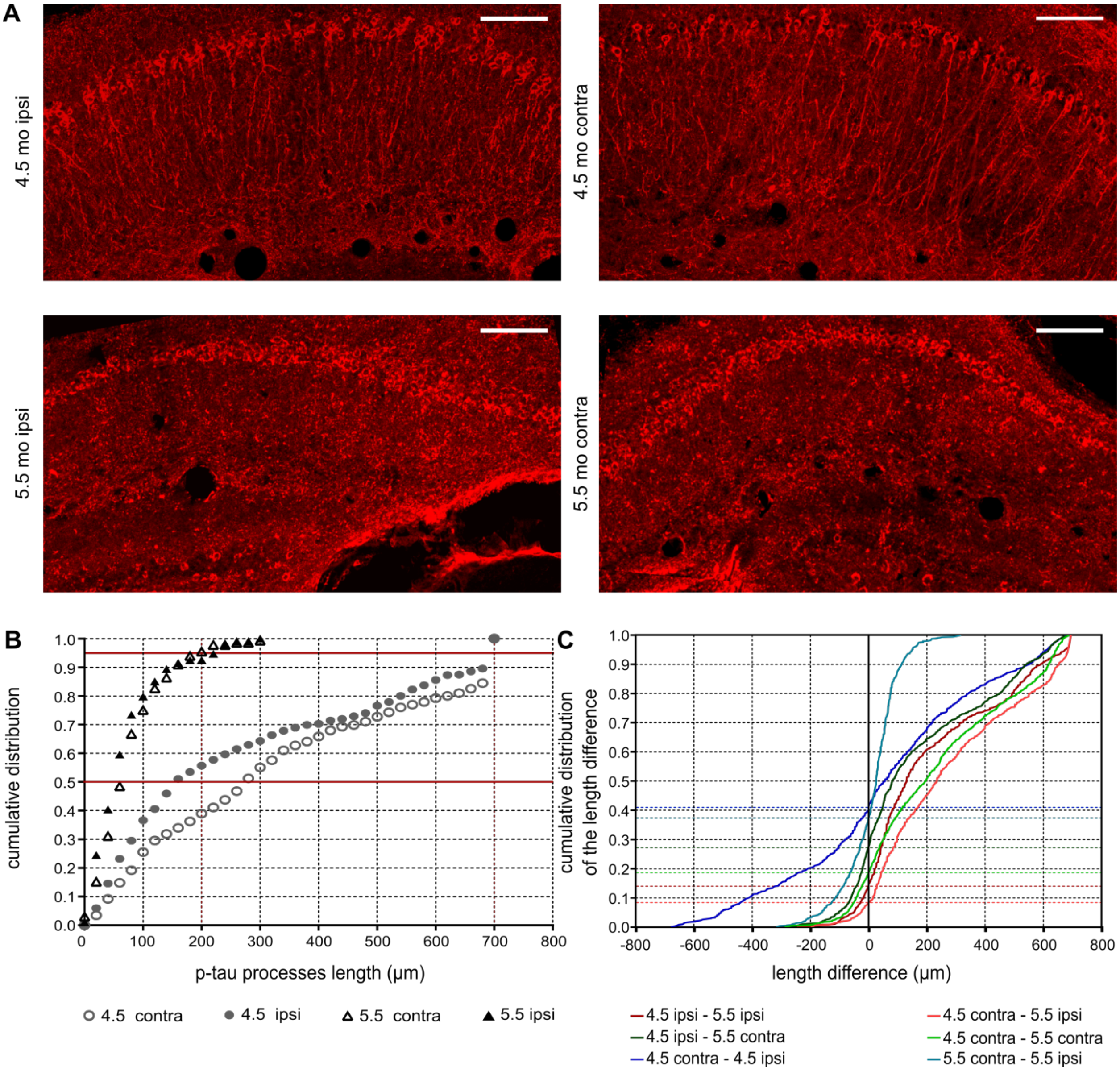


**Figure S2: A:** Phosphorylated tau signal emitted from the affected pyramidal neurons of the ipsilateral and contralateral side in the 4.5 and 5.5 months old brains. **B:** Comparison of the cumulative density function of the p-tau processes length as obtained for each group (4.5 mo contra, 4.5 mo ipsi, 5.5 mo contra and 5.5 mo ipsi). At the 95$^{th}$ percentile most of the p-tau processes in the older group are up to 300 µm long while 700 µm long in the younger brains. The differences are evident even at the 50$^{th}$ percentile. **C:** The cumulative density function of the p-tau processes difference in length obtained from the Monte Carlo simulation. Random samples from the probability density function of each group where subtracted and the cumulative density function of the difference was constructed. 91% (4.5 mo contra-5.5 mo ipsi) of the neurons on the younger contralateral side and 86% (4.5 mo ipsi-5.5 mo ipsi) of the neurons on the younger ipsilateral side are longer than those of the older ipsilateral side while 81% (4.5 mo contra-5.5

mo contra) and 72% (4.5 mo ipsi-5.5 mo contra) of the neurons are longer when comparing the younger contralateral and ipsilateral sides to the older contralateral side. The difference between the hemispheres in the 4.5 months brain is only 62% (4.5 mo contra-4.5 mo ipsi) and in the 5.5 months brain, which verifies the accuracy of the method as neuronal length should not differ. Scale bar, 100 μm.

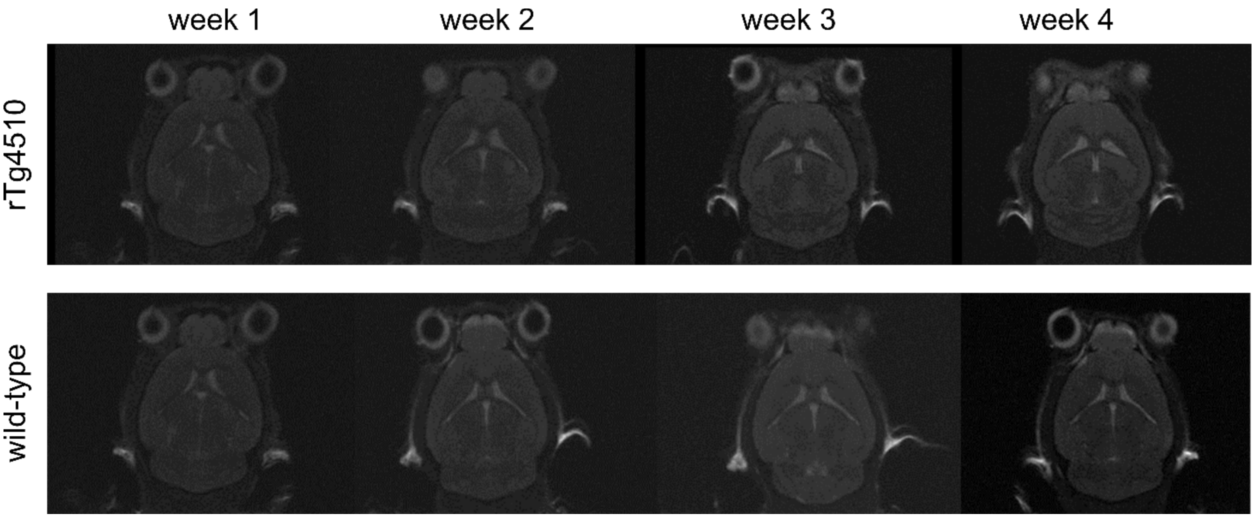


**Figure S3:** T2-weighted imaging was performed the day after sonication for four consecutive weeks. T2-weighted images did not show any hyperitensities indicative of edema occurrence.

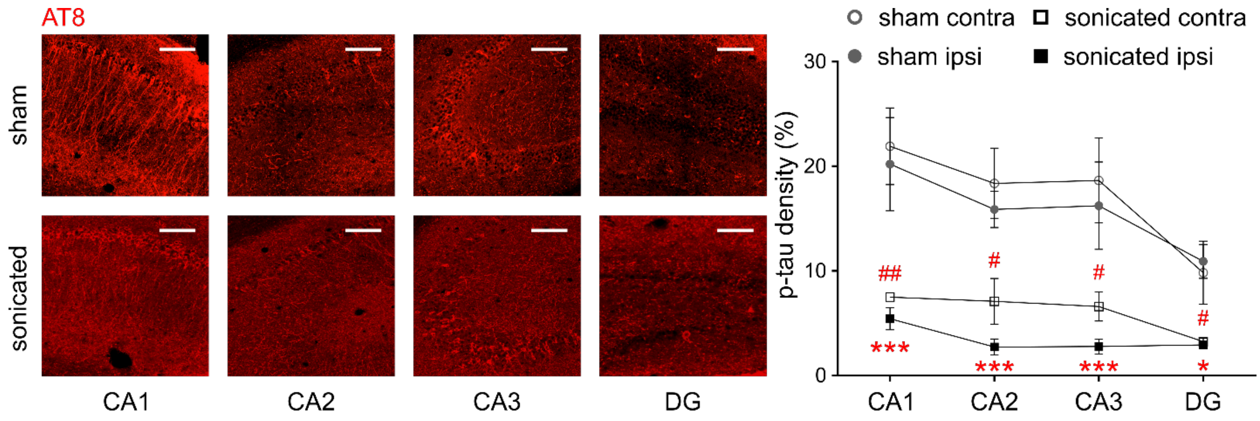


**Figure S4:** Signal distribution in the hippocampal subfields. Sections of the CA1, CA2, CA3 and Dentate Gyrus of the ipsilateral hemispheres of the sham and the sonicated brains are shown. CA1 and CA3 regions seem to be more affected by the pathology as shown by the increase in p-tau signal. On the other hand, the CA2 and the DG sectors appear to be less susceptible to the pathology at this age of the rTg4510 mouse line. The marker reduction in the ipsilateral side was at the order of 73.11% (P=0.0004), 82.86% (P=0.001), 82.98% (P=0.001) and 73.11% (P=0.03) in the CA1, CA2, CA3 and DG respectively when comparing the sham and sonicated brain, while 65.79% (P=0.001), 61.41% (P=0.008), 64.62% (P=0.005) and 67.34% (non-significant) when comparing the contralateral sides. The subfield distribution of the makers was similar across groups and hemispheres. Phosphorylated tau was significantly reduced in all the subfields of both hemispheres of the sonicated brains compared to the sham group with the ipsilateral side experiencing greater reductions. Scale bar, 100 μm.

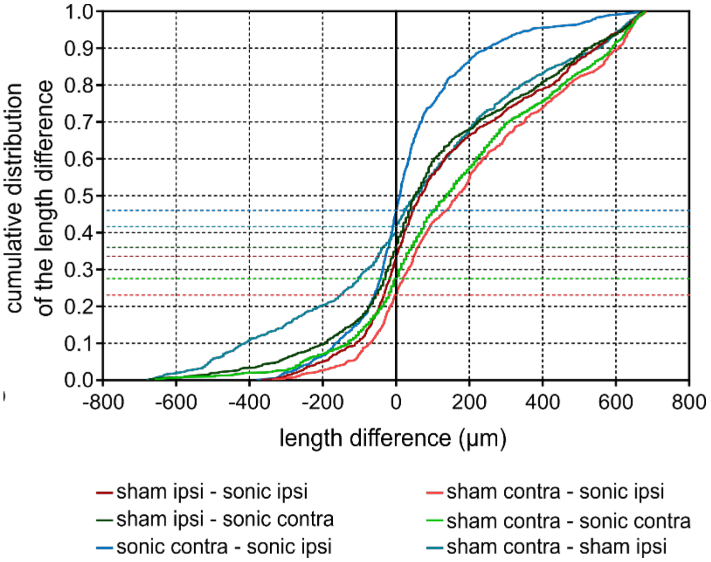


**Figure S5:** The cumulative density function of the differences in the p-tau processes length. The CDF for the length differences between the ipsilateral hemispheres of the two groups is shown in Figure 5C while all CDFs are presented here.
